# Thyrotoxicosis Associated with Ustekinumab Treatment for Psoriasis

**DOI:** 10.1155/2020/8868553

**Published:** 2020-08-18

**Authors:** Bayanne Olabi, Shanti Ayob

**Affiliations:** ^1^Department of Dermatology, Lauriston Building, Edinburgh EH3 9EN, UK; ^2^Department of Dermatology, NHS Treatment Centre, Nottingham NG7 2FT, UK

## Abstract

Biologic treatments have revolutionised the management of psoriasis in recent years; however, data on their safety profile in large populations and long-term effects are being gathered on an ongoing basis. Ustekinumab is a monoclonal antibody that targets interleukin-12/23 used in the treatment of moderate-to-severe psoriasis. Here, we report the case of a 32-year-old Caucasian gentleman who developed thyrotoxicosis following the commencement of ustekinumab treatment. Following control of thyroid status by the Endocrinology team, this recurred after recommencement of ustekinumab on two further occasions over a 5-year period. This is the second known reported association of this nature. Awareness of these possible adverse effects is imperative in managing patients and informing decision-making, and further long-term studies will help elucidate the precise safety profiles of biologic treatments.

## 1. Introduction

Psoriasis is a chronic immune-mediated skin condition driven by a complex interplay of effector cells and inflammatory cytokines [1]. Ustekinumab is a human IgG1_K_ monoclonal antibody that targets interleukin-12/23 used in the treatment of moderate-to-severe plaque psoriasis in adults. This is a report of a patient who developed three distinct episodes of thyrotoxicosis over the course of 5 years, each episode following the commencement and recommencement of ustekinumab treatment for psoriasis.

## 2. Case Presentation

A 32-year-old Caucasian gentleman with severe plaque psoriasis was commenced on ustekinumab therapy in March 2011 (45 mg, 3 monthly), following failure of treatment with narrowband UVB phototherapy, methotrexate, cyclosporine, etanercept, and adalimumab due to side effects and/or secondary failure.

On review in June 2012, he was noted to have a tremor, lid lag, and a moderate goitre. Biochemistry revealed thyrotoxicosis, with a serum T3 level of 30 pmol/l (normal range: 3.5–6.7 pmol/l), a T4 level of 74.6 pmol/l (10–19.8 pmol/l), and a TSH level of <0.1 mU/l (0.27–4.2 mU/l). He was commenced on carbimazole by the Endocrinology team and ustekinumab was stopped.

However, the patient wanted good control of his psoriasis before his wedding and wished to recommence ustekinumab, despite understanding the risks of uncontrolled hyperthyroidism. It was restarted in August 2012. Whilst on ustekinumab, his hyperthyroidism remained difficult to control over the next 2 years, with weight loss (5 kg within 3 months of restarting ustekinumab) and clinical hyperthyroidism (tremors, palpitations, palpable goitre), requiring multiple uptitrations of carbimazole. Ustekinumab was eventually stopped in June 2014 due to persistent uncontrolled hyperthyroidism and he was switched to a fumaric acid ester (Fumaderm), followed by acitretin, both of which were ineffective at controlling his psoriasis. Radioiodine therapy was successfully administered in June 2015, rendering him biochemically and clinically euthyroid.

The patient felt however that ustekinumab was the only treatment that satisfactorily controlled his psoriasis, and therefore after further discussion of potential risks this was restarted in August 2015. He developed hyperthyroidism again in November 2015, experiencing tremors and sweats with a serum T3 of 23.2 pmol/l, T4 of 43 pmol/l, and TSH of <0.1 mU/l. He required further treatment with carbimazole and eventually a second dose of radioiodine that caused hypothyroidism.

**Figure 1:**
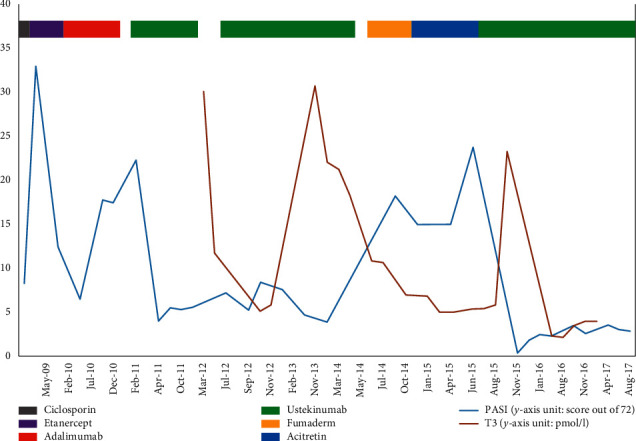
Graph to show PASI (blue solid line) and serum T3 level (solid pink line) during treatment with cyclosporine, etanercept, adalimumab, ustekinumab, Fumaderm, and acitretin between May 2009 and August 2017.

He remains on ustekinumab therapy to date and continues with long-term thyroxine replacement. The following chart displays the time-course of these events graphically (Figure 1).

## 3. Discussion

To our knowledge, this is the second reported case of ustekinumab treatment associated with thyrotoxicosis. In this case, the hyperthyroidism developed several months after first treatment with ustekinumab, and although the causative link was in question initially, recurrence of hyperthyroidism after recommencement of ustekinumab on two further separate occasions was more indicative of causality.

A previous case of autoimmune thyroiditis (Graves' disease) following ustekinumab treatment for psoriasis has recently been reported in a 68-year-old woman [2]. It was postulated that Graves' disease is mediated by Th2 pathways and that inhibition of Th1/Th17-mediated pathways by ustekinumab [3] may skew the Th1/Th2 balance, leading to the production of thyroid autoantibodies [4]. Other biologic treatments have been considered in this context, and two cases of autoimmune thyroid disease following treatment with anti-TNF-*α* agents have been previously reported in patients treated for rheumatoid arthritis [5, 6]. An article summarising 5-year single-centre experience with ustekinumab for psoriasis, which included 93 patients, reported a case of papillary thyroid cancer, but no reports of changes in thyroid state [7]. The British Association of Dermatologists Biologic Interventions Register (BADBIR) group collects data from most centres in the United Kingdom and Ireland and has also not reported any similar adverse reactions with ustekinumab in 450 patients [8]. There are no long-term data available about the safety profile of many biologic therapies in psoriasis as they have only been licensed relatively recently; therefore, awareness and reporting of these reactions are important in planning the management of these patients in the future.

## References

[1] Mahil S. K., Capon F., Barker J. N. (2016). Update on psoriasis immunopathogenesis and targeted immunotherapy. *Seminars in Immunopathology*.

[2] Lee S., An J., Kim D., Yoon M., Lee H. (2019). A case of interstitial lung disease and autoimmune thyroiditis associated with ustekinumab. *Acta Dermato Venereologica*.

[3] Koutruba N., Emer J., Lebwohl M. (2010). Review of ustekinumab, an interleukin-12 and interleukin-23 inhibitor used for the treatment of plaque psoriasis. *Therapeutics and Clinical Risk Management*.

[4] Ramos-Leví A. M., Marazuela M. (2016). Pathogenesis of thyroid autoimmune disease: the role of cellular mechanisms. *Endocrinología y Nutrición*.

[5] Vassilopoulos D., Sialevris K., Malahtari S., Deutsch M., Manolakopoulos S., Archimandritis A. J. (2010). Subacute thyroiditis presenting as fever of unknown origin in a patient with rheumatoid arthritis under etanercept treatment. *Journal of Clinical Rheumatology*.

[6] Van Lieshout A. W., Creemers M. C., Radstake T. R., Elving L. D., van Riel P. L. (2008). Graves’ disease in a patient with rheumatoid arthritis during treatment with anti-tumor necrosis factor-alpha. *The Journal of Rheumatology*.

[7] Vergou T., Moustou A. E., Antoniou C. (2017). Five-year experience with ustekinumab for psoriasis: real-life data of a single centre. *Journal of the European Academy of Dermatology and Venereology*.

[8] Warren R. B., Smith C. H., Yiu Z. Z. N. (2015). Differential drug survival of biologic therapies for the treatment of psoriasis: a prospective observational cohort study from the British Association of Dermatologists Biologic Interventions Register (BADBIR). *Journal of Investigative Dermatology*.

